# Supplementary material for: Novel Identification of Ankyrin-R in Cardiac Fibroblasts and a Potential Role in Heart Failure
Source: Int J Mol Sci. 2024 Aug 1;25(15):8403. doi: 10.3390/ijms25158403 (PMC11313496; doi:10.3390/ijms25158403)
Supplement: Supplementary file 1 [file ijms-25-08403-s001.zip › ijms-3092540-supplementary.pdf]

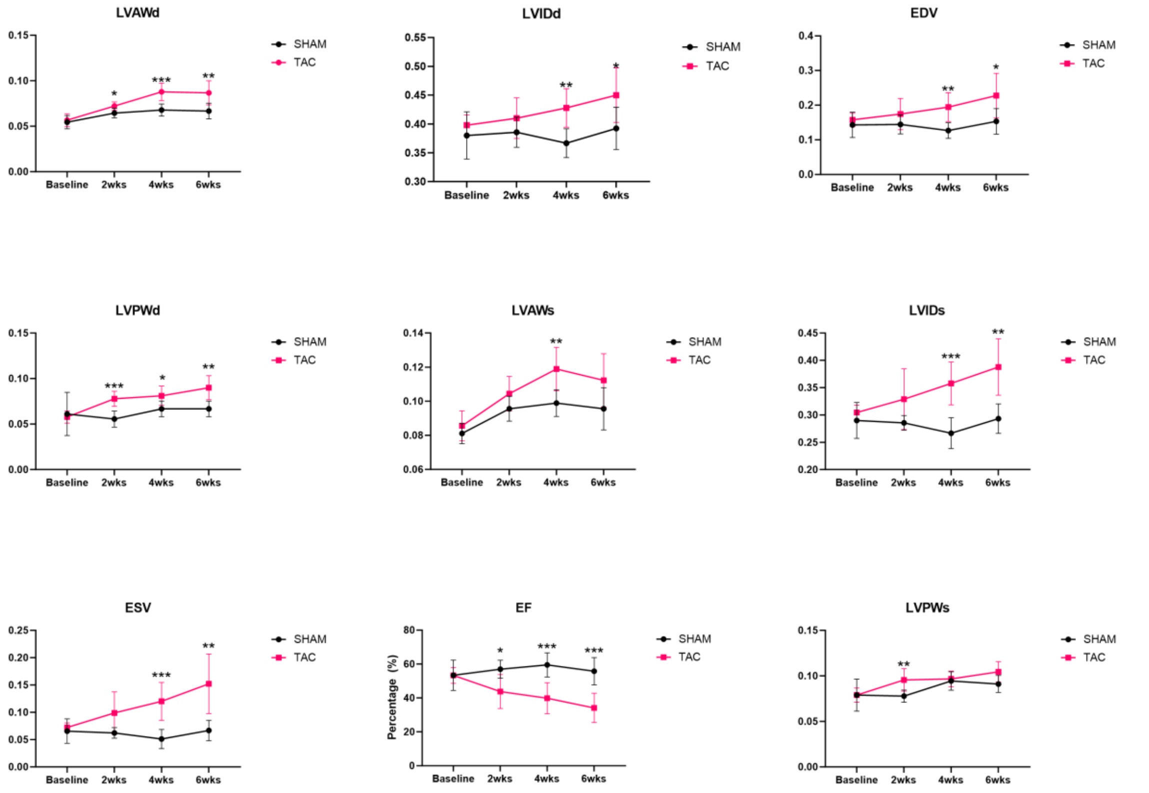

Supplementary Figure S1. Echocardiographic measurements of Sham and TAC surgery mice, showing decreased ejection fraction after 6 weeks. LVAWd (left ventricular anterior wall during diastole), LVIDd (left ventricular diameter during diastole), EDV (end-diastolic volume), LVPWd (left ventricular posterior wall thickness during diastole), LVAWs (left ventricular anterior wall during systole), LVIDs ((left ventricular diameter during systole), ESV (end-systolic volume), EF (ejection fraction), LVPWs (left ventricular posterior wall thickness during systole). Sham n = 9, TAC n = 10. Two-way ANOVA, mean ± SEM *p<0.05, **p<0.001, ***p<0.0001.

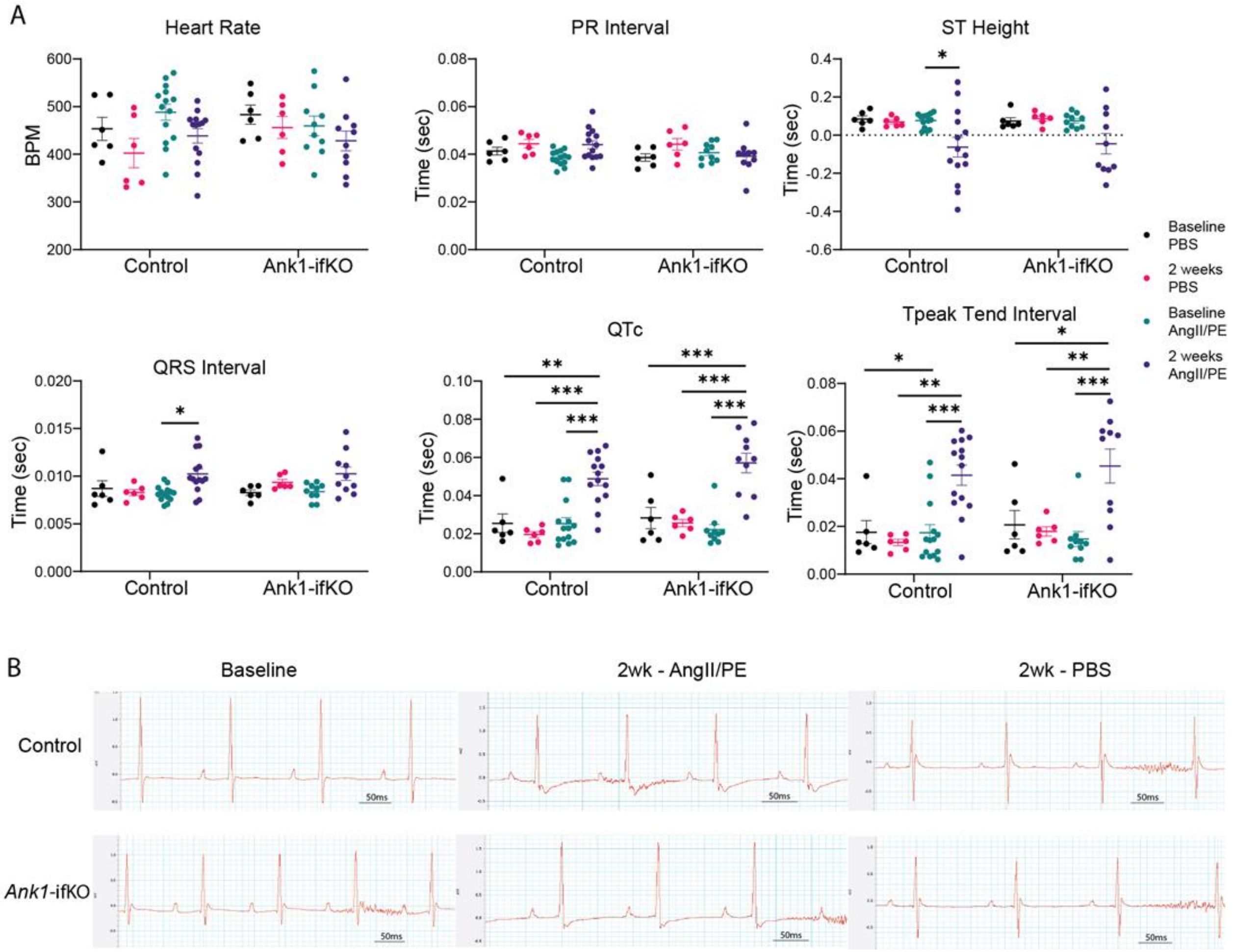

Supplemental Figure S2. Deletion of AnkR in activated fibroblasts protects against delayed depolarization and ST height elevation. **A)** Echocardiogram parameters recorded at baseline and after two weeks of AngII/PE treatment. AngII/PE treatment resulted in ST height elevation, T wave inversion, and prolonged QRS duration in control animals. Control PBS *n* = 6, Control AngII/PE *n* = 14; *Ank1*-ifKO PBS *n* = 6, *Ank1*-ifKO *n* = 10, 3-way ANOVA, mean ± SEM *p<0.05, **p<0.001, ***p<0.0001. **B)** Representative images of ECG recordings at baseline and after two weeks of treatment (AngII/PE or PBS). Scale bar = 50ms.
